# Views, Use, and Experiences of Web-Based Access to Pediatric Electronic Health Records for Children, Adolescents, and Parents: Scoping Review

**DOI:** 10.2196/40328

**Published:** 2022-11-22

**Authors:** Josefin Hagström, Charlotte Blease, Barbara Haage, Isabella Scandurra, Scharlett Hansson, Maria Hägglund

**Affiliations:** 1 Department of Women's and Children's Health Uppsala University Uppsala Sweden; 2 Beth Israel Deaconess Medical Center Harvard Medical School Boston, MA United States; 3 Department of Health Technologies Tallinn University of Technology Tallinn Estonia; 4 Informatics Örebro University School of Business Örebro Sweden

**Keywords:** electronic health record, patient-accessible electronic health record, adolescents, parents, children, patient experience, patient portal, electronic portal, review, scoping review, youth, patient perspective, user experience, patient access, mobile phone

## Abstract

**Background:**

Ongoing efforts worldwide to provide patients with patient-accessible electronic health records (PAEHRs) have led to variability in adolescent and parental access across providers, regions, and countries. There is no compilation of evidence to guide policy decisions in matters such as access age and the extent of parent proxy access. In this paper, we outline our scoping review of different stakeholders’ (including but not limited to end users) views, use, and experiences pertaining to web-based access to electronic health records (EHRs) by children, adolescents, and parents.

**Objective:**

The aim of this study was to identify, categorize, and summarize knowledge about different stakeholders’ (eg, children and adolescents, parents, health care professionals [HCPs], policy makers, and designers of patient portals or PAEHRs) views, use, and experiences of EHR access for children, adolescents, and parents.

**Methods:**

A scoping review was conducted according to the Arksey and O’Malley framework. A literature search identified eligible papers that focused on EHR access for children, adolescents, and parents that were published between 2007 and 2021. A number of databases were used to search for literature (PubMed, CINAHL, and PsycINFO).

**Results:**

The approach resulted in 4817 identified articles and 74 (1.54%) included articles. The papers were predominantly viewpoints based in the United States, and the number of studies on parents was larger than that on adolescents and HCPs combined. First, adolescents and parents without access anticipated low literacy and confidentiality issues; however, adolescents and parents who had accessed their records did not report such concerns. Second, the main issue for HCPs was maintaining adolescent confidentiality. This remained an issue after using PAEHRs for parents, HCPs, and other stakeholders but was not an experienced issue for adolescents. Third, the viewpoints of other stakeholders provided a number of suggestions to mitigate issues. Finally, education is needed for adolescents, parents, and HCPs.

**Conclusions:**

There is limited research on pediatric PAEHRs, particularly outside the United States, and on adolescents’ experiences with web-based access to their records. These findings could inform the design and implementation of future regulations regarding access to PAEHRs. Further examination is warranted on the experiences of adolescents, parents, and HCPs to improve usability and utility, inform universal principles reducing the current arbitrariness in the child’s age for own and parental access to EHRs among providers worldwide, and ensure that portals are equipped to safely and appropriately manage a wide variety of patient circumstances.

**International Registered Report Identifier (IRRID):**

RR2-10.2196/36158

## Introduction

### Background

Patients being enabled to read their health records on the web is a growing phenomenon. Patient-accessible electronic health records (PAEHRs) commonly include clinical information (eg, physician visit notes, laboratory test results, medications, diagnoses, and referrals), and enabling patients to access their electronic health records (EHRs) is thought to promote patient empowerment by involving patients in their own care [1]. The term *open notes* is often used to describe the specific practice of giving patients access to the free-text entries written by clinicians [2] and is considered an important part of any PAEHR. The websites that host PAEHRs, commonly developed by so-called EHR vendors, are often referred to as patient portals and, for the purposes of this study, *patient portals* will refer to tethered, secure websites that hold any type of health information recorded by a health care provider that users have access to. Today, health institutions in >15 countries are developing patient portals [3], and there is continuous adaptation of legal frameworks at a national level to improve use and ensure patients’ privacy [3,4].

An often-cited challenge to PAEHR implementation concerns how to manage access for parents, children, and adolescents [3]. The transfer of proxy access being managed by the parent or guardian (hereafter referred to as *parents*) into own access for the child is often conducted during adolescence, with the aim of protecting the adolescent’s privacy as well as to support the transition to adulthood. The need for protection arises as the individual begins seeking care for sensitive medical conditions such as mental health or reproductivity. The child’s need for autonomy in their relationship with their health care professional (HCP) is compromised during shared access with parents. So far, providers and countries have approached this dilemma in different ways. For example, the access age of the child varies, as well as when parents lose access and the age when patients gain self-access to their records. In some countries (eg, Finland and Estonia), parents are provided access (unless actively restricted by the child), whereas in other countries (eg, Sweden and Norway), parents are blocked from accessing records by law when their children reach a certain age threshold [5]. In France, adolescents receive access at the age of 18 years, when, in turn, the parents lose access. Decisions regarding earlier access in France can also depend on the perceived maturity of the minor. In many countries and regions, a lack of continuity in access to care is apparent [3]. In the United States, policies regarding age and privacy exceptions are dependent on state laws, which vary throughout the country. In 2021, the 21st Century Cures Act made it mandatory for health care providers to provide every patient with free electronic access to their clinical notes [6]. There is a possibility for withholding confidential information; however, questions still remain [7]. Evidently, the current lack of an international consensus on regulations for EHR access for parents, children, and adolescents has led to great variability.

The research of views, use, and experiences of PAEHRs to date has focused on HCPs and patients of the general adult population. The effects of PAEHRs are not conclusive, yet they indicate benefits including improved medication adherence and self-care as well as improved relationships between patients and their physicians [8-10]. However, a growing yet scarce body of literature is exploring access to EHRs for parents, children, and adolescents in particular. Although parents appreciate having access to their child’s records into adolescence [11], shared access to PAEHRs for parents and adolescents runs the risk of causing ethical dilemmas for HCPs. For example, some health information may be considered sensitive by adolescents, such as health care data pertaining to the disclosure of alcohol or drug abuse, sexual activity, or stigmatized illnesses such as anxiety or depression. Adolescents have also been observed to withhold information from HCPs if they are uncertain about who may access it [12,13]. With regard to adolescents’ self-access, it is thought that EHR access offers information transparency that might contribute to patient empowerment and enhanced health care; however, evidence suggests that the adolescent population requires targeted analysis. To date, one systematic review [14] has examined patient portals among pediatric patients. The review included only parents and adolescents and focused on empirical studies, and 10 of the 11 studies were based in the United States. Mostly positive feedback was found; however, there was some concern about medical literacy and its effects on the communication between adolescents, parents, and HCPs.

### Study Objectives

The objective was to identify, categorize, and summarize knowledge about different stakeholders’ (eg, children and adolescents, parents, HCPs, policy makers, and patient portal designers) views, use, and experiences of PAEHR access for children, adolescents, and parents. The findings will aid policy makers in designing future regulations regarding EHR access for parents and adolescents and potentially improve the design and implementation of PAEHRs to meet the needs of the end users. The concept “view” refers to attitudes, expectations, and thoughts; “use” refers to portal feature use and use rates; and “experience” includes experiences pertaining to, for example, satisfaction, concerns, and literacy. We use the definition of Davis et al [15] for a scoping review—“a synthesis and analysis of a wide range of research and nonresearch material to provide greater conceptual clarity about a specific topic or field of evidence”—with the adjustment of not including nonresearch material because of restrictions of the study search strategy. We defined *policy maker* as an agent with capacity or responsibility for deciding policies on PAEHRs (either national, regional, institutional, or as an HCP). The following research question was examined in detail: how do different stakeholders experience children’s, adolescents’, and parents’ web-based access to the EHRs of children and adolescents? With regard to experiences of HCPs and HCP experts (among other stakeholders) who document in the records or manage the records within their professions, we focused on how these individuals perceive or are affected by the situation where children, adolescents, and parents have access to the EHRs of children and adolescents.

## Methods

### Scoping Review Approach

The full protocol for this review has been published previously [16]. To summarize, a literature search on PAEHRs for children, adolescents, and parents was conducted using the Arksey and O’Malley [17] framework. The framework includes six stages: (1) identifying the research question; (2) identifying relevant studies; (3) study selection; (4) charting the data; (5) collating, summarizing, and reporting the results; and (6) consulting with relevant stakeholders. To ensure reproducibility and traceability, the scoping review was conducted according to the PRISMA (Preferred Reporting Items for Systematic Reviews and Meta-Analyses) extension for Scoping Reviews (PRISMA-ScR) checklist to report our results (Multimedia Appendix 1) [18].

### Stage 1: Identifying the Research Question

Our research question was as follows: how do different stakeholders experience children’s, adolescents’, and parents’ web-based access to the EHRs of children and adolescents?

### Stage 2: Identifying Relevant Studies

A comprehensive literature search was conducted on June 23, 2021, by an experienced research librarian at Uppsala University, who provided the research team with the results immediately after conducting the search. The search included the following electronic literature databases: PubMed, CINAHL, and PsycINFO. The search included peer-reviewed literature published between 2005 and September 2021, where the year 2005 was chosen as a cutoff as we expected to not identify any relevant publications on pediatric PAEHRs before this. Search terms were identified using input from the research team and the literature. The references of the identified articles were scanned backward to identify prior work to consider for the research topic. The search query with Boolean operators was presented in the published protocol [16].

### Stage 3: Selecting Eligible Studies

The inclusion and exclusion criteria were informed by the review process and were applied at the study selection stage.

#### Inclusion Criteria

Studies were included if they met the following criteria: (1) the patient user population was children, adolescents, and parents; (2) the population studied was children, adolescents, parents, HCPs, and other stakeholders; (3) outcomes were views, use, or experiences of access or proxy access to PAEHRs; and (4) the study design was all study types.

We defined patients aged ≤12 years as children, patients aged 13 to 18 years as adolescents, and those aged ≥18 years as adults. However, to increase the number of eligible studies for the adolescent population, the age of 19 to 20 years was included if a study participant group included a majority of adolescents (eg, aged 15-19 years).

#### Exclusion Criteria

Studies were excluded if they (1) were not written in English, (2) were published outside the study period, or (3) did not focus on pediatric PAEHRs.

#### Search Strategy

The search results were imported into the software program Rayyan (Rayyan Systems Inc) [19] according to the following headings: publication type, publication year, country, sample characteristics, setting, study aim, research question, and conclusions. Duplicates were removed. Titles and abstracts were screened by the authors with consideration of the eligibility criteria. The articles were divided between the investigators (excluding IS) so that each article was screened by at least 2 people. Any disagreements were resolved through group discussion and, if needed, with the addition of a third reader.

### Stage 4: Charting the Data

The first author set up a Microsoft Excel spreadsheet to which all researchers added information independently, including the following study characteristics: reference ID, type of identification, title, authors, year, journal, type of publication, study design, participant description, country, treatment setting, clinical field, research question, and main conclusions. The first author held the main responsibility for verifying the accuracy of the data (Multimedia Appendix 2 [11,20-92]). If the abstract and title were insufficient for assessment, the full text was screened. Multiple authors could provide an assessment of the same paper, and instances of disagreement were resolved through discussion. In the second stage, full-text papers were evenly assigned to 2 authors. Instances of disagreement were resolved through discussion and sometimes by bringing in a third reader. The ideas that emerged during the process were discussed among the authors in regular meetings set up by the main author.

### Stage 5: Collating, Summarizing, and Reporting the Results

The results reported in the included studies were compiled and read multiple times. In the Microsoft Excel spreadsheet, papers were categorized according to the stakeholder group studied: children and adolescents, parents, HCPs, or expert viewpoints. In total, 2 students categorized the viewpoints into three groups: (1) experts, such as HCPs, IT experts, or researchers; (2) policy makers; and (3) public opinion. In a meeting, 2 authors were assigned to each stakeholder group through discussion, where the first of the following authors listed was mainly responsible: children and adolescents were assigned to JH and BH, parents were assigned to MH and SH, HCPs were assigned to CB and IS, and viewpoints were assigned to JH and MH (as first and senior author, respectively). The results from the included studies were then independently analyzed and jointly drafted in a shared Google Docs. For organization of the results, key themes were adapted from a previous scoping review of the literature on PAEHRs in mental health [93]. These were refined by the main author using thematic analysis [94]. During this process, the material was gathered according to themes, and themes were reviewed and defined. This synthetization of results was conducted primarily by the main author but was discussed in research team meetings.

### Stage 6: Consultation

To gain further insights on the topic, the results were shared with stakeholder representatives, including a pediatric oncologist, members of a young patient council at a public hospital in Sweden, and the Ombudsman for Children in Sweden. The representatives were provided with material via email and invited to choose to provide their thoughts in text via email or verbally during a web-based meeting.

## Results

### Study Selection

**Figure 1:**
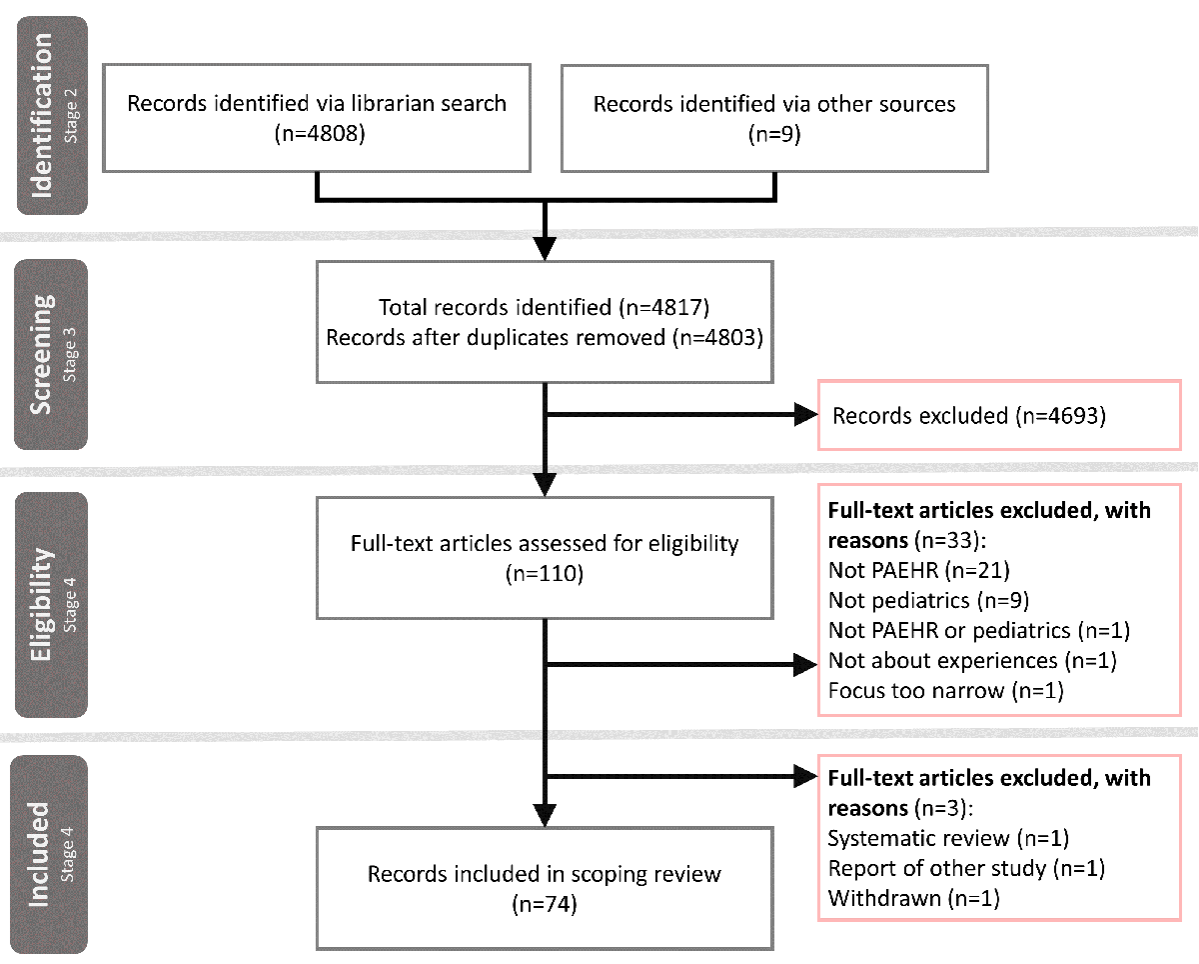
PRISMA (Preferred Reporting Items for Systematic Reviews and Meta-Analyses) flow diagram adapted from Moher et al [22]. PAEHR: patient-accessible electronic health record.

Figure 1 shows the study selection process in a PRISMA diagram [95]. In total, 4817 records were identified, of which 4808 (99.81%) were identified via a database search and 9 (0.19%) were identified via other sources. After removing duplicates, 99.71% (4803/4817) of the records remained for screening of abstracts, titles, and keywords. In this process, 97.71% (4693/4803) of the records were excluded, resulting in 110 full-text articles to be assessed for eligibility. As a result of this, 1.6% (77/4817) of the total records identified met the inclusion criteria. During the analysis, 0.06% (3/4817) of the records were excluded, leaving 1.54% (74/4817) of articles included in the review.

### Basic Characteristics of the Body of Evidence

**Figure 2:**
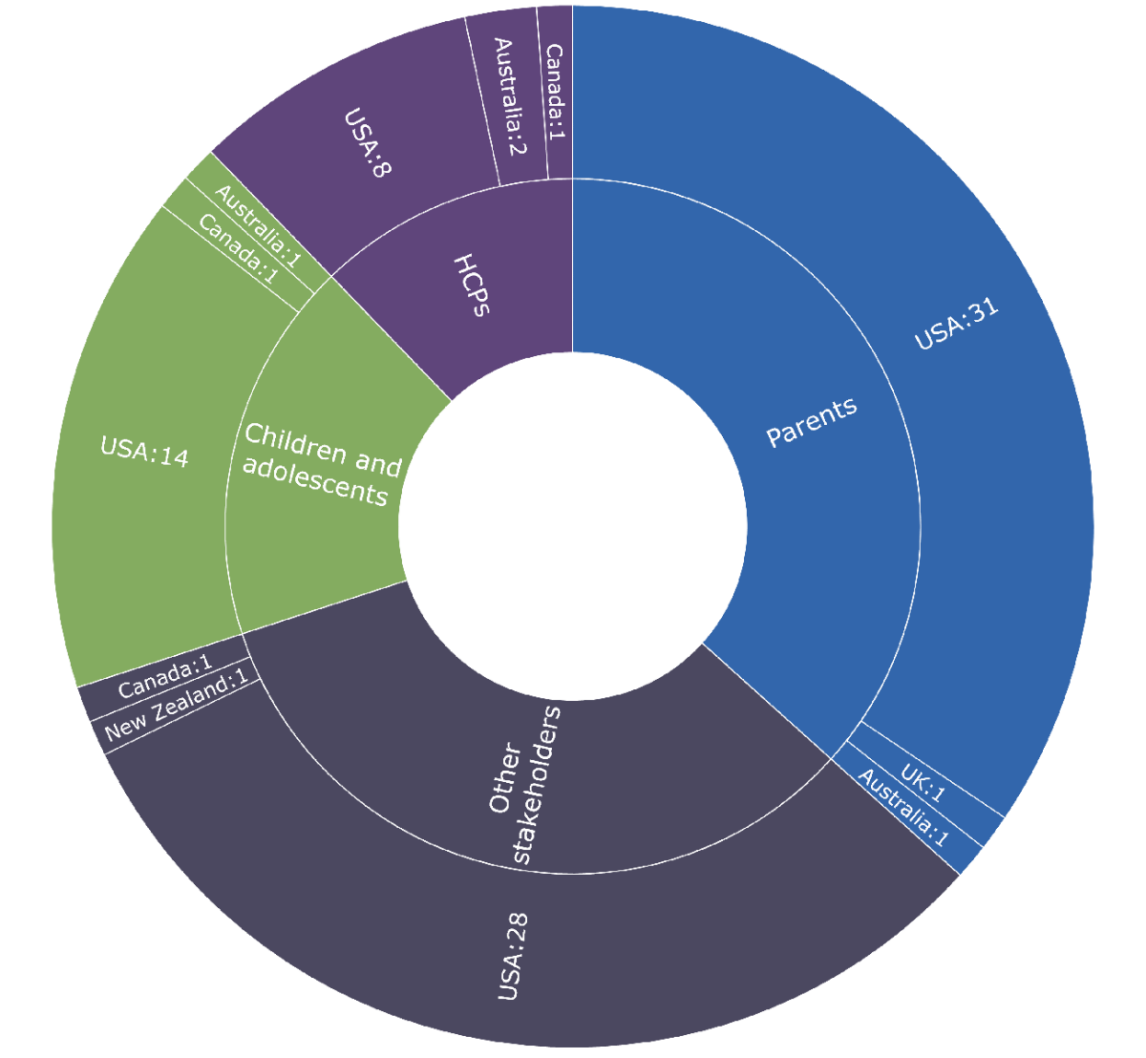
Included publications by country and studied stakeholder group. HCP: health care professional; UK: United Kingdom; USA: United States of America.

**Figure 3:**
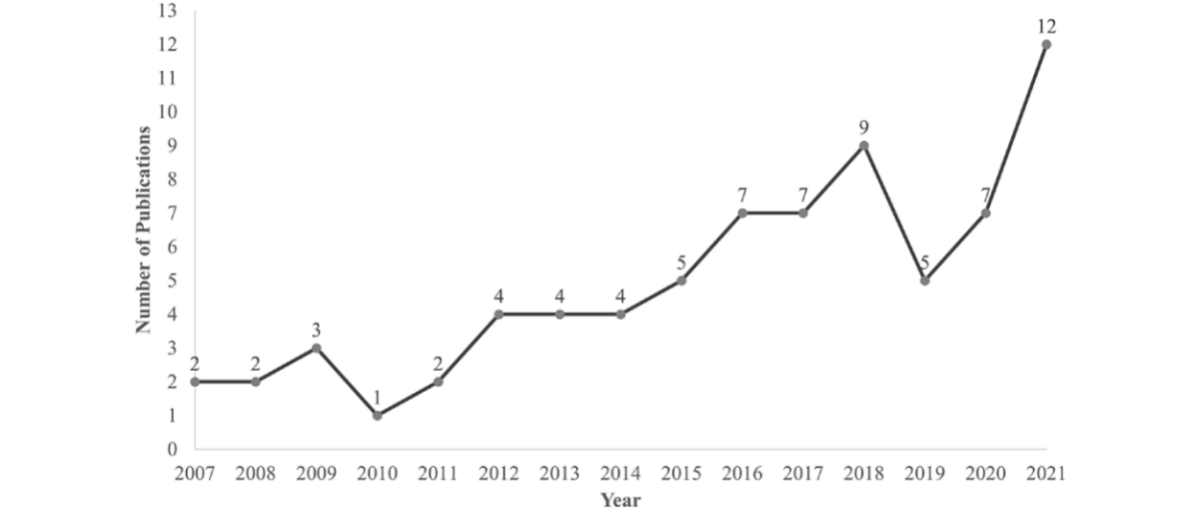
Number of publications by year.

The included studies were mainly viewpoint papers or used quantitative methods (Table 1), and 92% (68/74) were based in the United States (Figure 2). The number of articles published in the area of PAEHRs for parents, children, and adolescents was fairly stable over time (Figure 3), ranging from 3% (2/74) of the articles in 2007 to 16% (12/74) in 2021. An increase can be observed for 2018 and 2021, and none of the articles during these years belonged to a special issue.

**Table 1:** Basic characteristics of the included studies (N=74).^a^

Parameter		Total, n (%)
**Study design**		
	Viewpoint or comment	27 (36.5)
	Quantitative	27 (36.5)
	Qualitative	13 (17.6)
	Mixed methods	7 (9.5)
**Publication year**		
	2007-2009	7 (9.5)
	2010-2012	7 (9.5)
	2013-2015	13 (17.6)
	2016-2018	23 (31.1)
	2019-2021	24 (32.4)
**Country**		
	Australia	3 (4.1)
	Canada	1 (1.4)
	New Zealand	1 (1.4)
	United Kingdom	1 (1.4)
	United States	68 (91.9)
**Study participants^b^**		
	Children and adolescents	6454 (5.5)
	Parents	110,184 (94.1)
	Health care professionals	496 (0.4)
	N/A^c^ (no participants or not specified; studies)	34 (45.9)
**Treatment setting**		
	Pediatric	34 (45.9)
	Adolescent	15 (20.3)
	Adult	2 (2.7)
	Inpatient	15 (20.3)
	Outpatient	20 (27)
	Academic	1 (1.4)
	N/A	7 (9.5)
**Clinical field**		
	Chronic illnesses (cystic fibrosis, juvenile idiopathic arthritis, or diabetes mellitus)	6 (8.1)
	Psychiatry	4 (5.4)
	Intensive care	4 (5.4)
	Gastroenterology	2 (2.7)
	Hematology	2 (2.7)
	Obstetrics and gynecology	2 (2.7)
	Neonatal care	2 (2.7)
	Cancer	1 (1.4)
	Cardiology	1 (1.4)
	Pulmonology	1 (1.4)
	Emergency	1 (1.4)
	Hepatology	1 (1.4)
	Subspeciality	1 (1.4)
	Radiology	1 (1.4)
	N/A	7 (9.5)

^a^Individual papers can be assigned to various subparameters at the same time, which means that percentage totals of >100% can be achieved.

^b^The number of study participants was accumulated based on empirical and observational studies that included a reported number of study participants.

^c^N/A: not applicable.

### Search Results

The results were divided into four groups of stakeholders: (1) children and adolescents, (2) parents, (3) HCPs, and (4) other stakeholders. For children, adolescents, and parents, the identified categories were adoption and use, positive views and experiences, and concerns and negative experiences. For HCPs, the identified categories were positive views and experiences and concerns and negative experiences. For expert analysis or viewpoints, the identified categories were positive views and experiences and concerns.

### Children and Adolescents

#### Overview

Views, use, or experiences of PAEHRs among children and adolescents comprised a relatively small part (16/74, 22%) of the included studies. Of these 16 studies, 14 (88%) were conducted in the United States, 1 (6%) was conducted in Australia [20], and 1 (6%) was conducted in Canada [21]. Most of these studies were observational (6/16, 38%), followed by surveys (4/16, 25%), qualitative studies (focus groups or interviews; 3/16, 19%), and mixed methods studies (2/16, 12%). Only 1 opinion paper was included, authored by a male patient aged 15 years [22]. Survey studies ranged from 20 [23] to 1006 [21] participants. Qualitative studies used focus group interviews (2/16, 12% of the studies) [20,24] and individual interviews (1/16, 6% of the studies) [25]. The most frequent care settings were pediatric inpatient care, primary care, psychiatry, and nonclinical care. In total, 12% (2/16) of the studies focused on the general population [20,21]. Observational studies focused on adoption and use over time, demographic data, and frequently used functions of patient portals [26-31], whereas survey studies explored satisfaction with reading the records [23,32,33], literacy [23,32], intervention effects [33], attitudes toward web-based patient portals [21,34], and barriers to adoption [34]. The studies included adolescents aged between 12 and 20 years, and 12% (2/16) of the studies included patients aged ≥18 years [21,32]. A few studies (2/16, 12%) included adolescents and proxy users and did not distinguish adolescent patients from proxy users in their analyses [26,31].

#### Adoption and Use

A number of studies (4/16, 25%) reported low adoption and use of patient portals among adolescents compared with other age groups [27-29,31]. In a US study, 11% of patients aged 10 to 17 years had activated an account at a patient portal implemented 3 years before [27]. Similarly, a study that allowed for surrogate access and individual accounts for patients aged >13 years with parental consent found that adolescent patients composed 16.5% of all log-ins, although use increased during late adolescence [29]. A study based in Canada identified an age-related difference where younger adolescents (aged 12-15 years) were more open than older adolescents (aged 16-19 years) to sharing their notes with parents [21]; however, a US study with a smaller sample size observed a similar tendency but no significant difference [33]. For adolescent patients with cancer, the perceived value of record access decreased during recovery [35]. Knowledge of PAEHRs was reported as low not only among adolescents without access to a patient portal in US studies [21,24,34] but also in a focus group study based in Australia where adolescents had access to their EHR from the age of 14 years [20]. The studies were inconclusive on gender differences in adolescents’ PAEHR adoption, finding either no differences [30,33] or a greater inclination among female patients [27]. In 6% (1/16) of the studies, male patients aged between 12 and 17 years had the lowest percentage of viewing their results in the patient portal (<1%) [28]. A study of 96 urban, low-income African American late adolescents in outpatient care found that male patients were more likely than female patients (*P*=.001) to consider allowing proxy access [33]. Regarding mode of access, adolescents in 12% (2/16) of the studies reported a preference for smartphones or tablet devices over computers [34,35].

#### Positive Views and Experiences

Studies that explored views on PAEHRs among adolescents who had not previously accessed their records identified a strong interest in access [20,21,24,33,34]. For example, among 1006 adolescents, 84% supported the idea that adolescents should be able to read their records on the web [21]. Adolescents wished to receive information about EHRs from HCPs according to their future needs [20,24]. Notably, an intervention study in which adolescents in primary care were informed about a patient portal observed an increase in portal account activation but not in use [33].

Positive expectations were confirmed by adolescents reading their records, with high satisfaction reported by studies in gastroenterology (9.2/10) [32], psychiatry (8.8/10) [23], and primary care (79%) [33]. In the study by Hong et al [35], adolescents with cancer and blood disorders read their records to ensure accuracy and check for updates. For these adolescents, reading their records led to reduced anxiety, enhanced knowledge about their illness, an ability to ask informed questions, and more reflection on their health. If needed, they consulted the internet or asked their parents. A US study conducted in a psychiatric ward found that having record access led patients’ trust in their health provider to either increase or remain the same [23]. In total, 12% (2/16) of the studies observed adequate literacy, with almost no exceptions among patients in psychiatry [23,32]. Both adolescents with and without experience of having access to their records foresaw empowerment [22,24,25,35]; a male patient aged 17 years stated in an opinion paper [22] that access “could help my generation learn about our health care system” and “encourage [adolescents] to take more responsibility for our health.” Patients with cancer anticipated that PAEHRs could support the transition from pediatric to adult care [35]. A high school senior in hematology who had used a patient portal suggested that the records could be jointly managed by themselves and their parents during the transition to adult care [25].

Better recall was an anticipated benefit among adolescents who did not access their records [20,22]. Furthermore, adolescents who did not have access to their records foresaw the utility of checking test results [21,24,34], messaging [20,24], viewing medications [20,21,34], reading visit notes [20], reviewing appointments [21,24], and viewing allergies [24]. In primary care contexts, adolescents valued being able to ask questions via email rather than in person, particularly concerning sensitive information [24]. Similarly, the most accessed information in observational studies was commonly test results [27,30,35], messaging [27,31,35], appointments [27,30,35], and medications [30,35]. Reminders were considered useful for planning around daily life [25,33,35], and a frequently asked questions section was suggested for ease of use [24].

Adolescents with cancer or a blood disorder who had accessed their records reported no concerns about what their parents would see in the EHR [35]. In an institute providing primary and mental health care that used a patient portal where a minor’s consent was required when aged >10 years to release information to parents, HCPs had received no complaints about confidentiality from adolescents since the implementation [27]. In an Australian focus group study, a participant noted that, in spite of valuing privacy, timely access to medical data in a critical situation was more meaningful [20].

#### Concerns and Negative Experiences

Although relatively few, the leading concern was health literacy among adolescents. Adolescents without access to their EHRs expressed worry about not being able to understand and appropriately interpret the information in the EHRs [21,24]. Among patients in psychiatry who read their records, half reported not understanding the discharge criteria [23]. In studies where adolescent patients had the option to suggest note changes, edits concerned personal history and anthropometrics [23] as well as allergies and medication reconciliation [32].

Concerns about internet security and confidentiality whereby parents might access their EHR were expressed by adolescents with no access to their records and who were patients in an outpatient or nonclinical setting [21,24]. A teenager in another study suggested that the relationship with the parent may affect the teenager’s feelings toward parental access, and in case of shared access, a private email option would be useful [25]. Adolescents without EHR access reported feeling uncomfortable with sharing their health information on social media [21].

### Parents

#### Overview

Parents’ and guardians’ experiences with web-based access to health records comprised more than a third of the studies (33/74, 45%). Of these 33 studies, 31 (94%) were conducted in the United States, 1 (3%) was conducted in Australia [36], and 1 (3%) was conducted in the United Kingdom [37]. The most common studies were surveys (10/33, 30%), followed by qualitative (9/33, 27%), observational (9/33, 27%), and mixed methods (5/33, 15%) studies. Among these were an opinion piece coauthored by a parent [25] and a usability test where 16 parents evaluated the usability of a patient portal prototype [38]. The most common settings were pediatric inpatient care, outpatient care, in-hospital care, primary care, congenital cardiac surgery, and hematology. The observational studies focused on adoption and use over time, demographic data, and frequently used functions of patient portals. The qualitative studies included both individual interviews (5/33, 15% of the studies, whereof 1/5, 20% also included observations) and focus group interviews (3/33, 9% of the studies). The survey studies ranged from 25 [39] to 3672 [40] participants. A total of 12% (4/33) of the studies had <100 participants, and only 6% (2/33) of the studies had >500 participants. Of the survey studies, 12% (4/33) explored parents’ thoughts about using a web-based patient portal [41-43] or their teenagers using such a portal [44] in the future. Of the remaining 8 survey studies, 3 (38%) focused specifically on errors in the record and patient safety issues [26,40,45]. In total, 6% (2/33) of the studies did not distinguish between parents and patients in their analyses [40,45]**.**

#### Adoption and Use

The studies reported high rates of patient portal adoption and use among parents during the first years of the child’s life [29,30]. In both Australia and the United States, studies identified the highest rates of patient portal activation for the youngest children of both sexes aged 0 to 11 [28] and 0 to 14 years [36]. In studies that required the assent of older adolescents for parental access, parents’ use of patient portals decreased. A study of a patient portal that required such assent received no applications for unrestricted access, and 80.4% of parents or guardians who enrolled had children aged <10 years [27]. In a longitudinal study where there were no restrictions, 93.62% (16,036/17,128) of all pediatric patients during the study period had a surrogate (parents or legal guardians), and surrogate users accounted for 83.2% of all log-ins for adolescent patients [29]. There was higher use among parents of children with chronic diseases [46]. Another study observed a 100% adoption rate among parents as proxy users for children aged 0 to 11 years, whereas merely 5.9% of parents of adolescents enrolled [30].

In an inpatient setting, a study [47] found that 27.89% (530/1900) of families created a patient portal account, 47.8% (238/498) used the portal within 3 months of registration, and 15.9% (79/498) continued using the portal 3 to 6 months after creating the account. A US study identified disparities in social demographics; parents who identified as Hispanic, Asian, or “other races” than White were less likely to use a patient portal, which was hypothesized to be related to language barriers and device accessibility [48]. The same and another study identified that privately insured parents were more likely to enroll in portal activation than those with public insurance [46,48]. In a study in which 12 children died during the study period, most families continued accessing their children’s records after their death [49]. A study of parents of children with attention-deficit/hyperactivity disorder found that, although half of the participants used their home computer to read the records, one-third accessed the portal on their smartphone and that barriers to use included lack of awareness, lack of internet access, lack of time, and password problems [50]. Schneider et al [37] identified four different use styles families at a children’s hospital in the United Kingdom applied to access the children’s records: controlling (approach-oriented and highly motivated to use PAEHRs), collaborating (approach-oriented and motivated to use PAEHRs), co-operating (avoidance-oriented and less motivated to use PAEHRs), and avoiding (very avoidance-oriented and not motivated to use PAEHRs).

#### Positive Views and Experiences

Several studies (4/33, 12%) focused on parents’ expectations or thoughts about PAEHR use before actually having experienced access to their child’s EHR [24,42,51,52]. In a 2013 US study, parents were approached in the waiting room and given a demonstration of the patient portal. A total of 72% (46/64) of the participants had not heard of the patient portal before, and only 28% (5/18) of those who had heard of the portal had used it. Nearly 70% (44/64) of the parents intended to use the patient portal after the demonstration [42]. Expectations were mostly positive and confirmed by studies with parents who had experience of record access, yet concerns were also discussed, which will be presented in the following section.

Better recall or reinforcement of information was reported as a benefit in many studies (7/33, 21%) [24,38,45,51-54], as was improved parental knowledge and understanding of their child’s health [39,51,53,55-59,96] and a sense of control [39]. In addition to access to information, parents reported enhanced communication and partnership with providers [11,39,45,51,53,55-58]. In a study on parents of hospitalized children, the addition to the PAEHR of pictures of staff taking care of the child was highly appreciated [58]. Another reported benefit was not having to bother clinicians [56,57,96]. As anticipated by parents of hospitalized children [51], having access to the child’s record also improved parental empowerment and the parents’ ability to advocate for their child [11,43,53,55,56]. Furthermore, parents of children with cancer or chronic illnesses described reduced anxiety as a positive result of having access to their children’s records [11,96]. The benefit of error detection was both reported by parents who had experiences of accessing their child’s records [35,55,56,58] and anticipated by those who did not [51].

Records were used to prepare for discussions with clinicians [35,39,56], formulate questions, and ask for explanations [35,56]. Another study observed that parents used the portal to ask questions about their children’s minor illnesses and request medication refills [27]. In studies in which parents were asked for suggestions for portal improvement, they often cited more information, such as a portal use tutorial [56], more educational links and resources [57,58], medical explanations or interpretations [38], and clarification of medical jargon [38]. However, in a survey study of 25 parents with real-time access to their children’s EHR, none considered notes more confusing than helpful [39].

Studies varied in the available portal features and details of reporting use. In total, 6% (2/33) of the studies provided a similar broad functionality, consequently seeing a similar use where one study [30] found high use of appointment reviews (85%), messaging (84%), test results (79%), and immunizations (79%) and the other [46] found parents to frequently access immunizations (80%), messaging (72%), appointment reviews (55%), and test results (50%).

Parents of children who were seriously ill consistently reported positive experiences, for example, parents who had immediate access to laboratory test results in an inpatient portal during a child’s hospitalization [55] and parents of children diagnosed with cystic fibrosis, juvenile idiopathic arthritis, or diabetes mellitus [11]. Chung et al [43] reported that 92% (78/85) of parents of hospitalized neonatal children wished to receive information even if it was “bad news.” A study among parents of patients in pediatric radiology found that, although only 12.1% (18/104) accessed a web-based portal to check their children’s test results, 65% prioritized minimal waiting time as the most important aspect for receiving results [60].

Some studies explored parents’ views on their teenagers accessing their own records, and parents saw it as a way for the teenagers to take better control of their own health care [24,96]. When parents of adolescents in juvenile detention were asked for their opinion on giving their teenagers access to their health records, 70% were positive, and 100% felt that the adolescent should be able to share this information with their parents through the web-based system [61]. Parents also felt that the PAEHR would be useful when transitioning to adult care or another care provider [35,51].

#### Concerns and Negative Experiences

Before having access to the record, parents worried about information being released without face-to-face communication [51,53]. When it came to adolescents having access to their own records, parents had privacy concerns that the portal might be hacked [61], that the teenager would be pressured to share information [61], or that billing of confidential services would cause privacy breaches [24]. Some requested that parents be required to consent to teenagers having access to portals [24,61] and were worried that teenagers would make appointments without parents knowing and wanted to be informed about email conversations [24]. Moreover, parents worried that adolescents might not reveal sensitive information if they knew it would be visible to their parents [51]. In a US survey study of 93 parents where half were parents of adolescents, 68% were negative about their children receiving information from their HCP through a secure web portal [44]. In a study in which parents of children in an intensive care unit were provided with real-time EHR access on a large monitor, parents expressed concern about visibility to bypassers [56]. Issues around parents’ loss of access to the record as the child enters adolescence were highlighted by Carlson et al [25], suggesting that record access needs to be an integrated part of the transition from childhood through adolescence and into adult health care. In the study by Hong et al [35], it was found that parents of teenagers with cancer would act as intermediaries in communication with HCPs as teenagers preferred to discuss their health with their parents rather than with clinicians. Thus, proxy access was considered essential. Parents in this study also expressed concerns about negative results being immediately available to teenagers, worrying that they might cause anxiety [35].

Some felt that teenagers may not understand all medical information, including test results [24], and that they might use the portal inappropriately and would need education [24,25]. Medical jargon was reported as an expected challenge in several studies (4/33, 12%) [38,43,51,96] as well as not being able to interpret complex results without context or explanation [56]. Parents of teenagers with cancer reported searching on the web to help make sense of the medical record and seeking additional information not readily available on MyChart [35]. Among 270 parents in a pediatric outpatient setting, 52.5% expected to read the medical records if they had access to them, with one-third indicating that they “sometimes” needed help reading health materials [41]. In another US study, 5% of surveyed parents of children with cancer reported understanding the notes to be “somewhat” or “very difficult” [59]. However, a study found that, among patients and families finding a serious mistake in visit notes, only approximately half reported the mistake, barriers including lack of knowledge of how to report but also fear or retribution [40].

Among concerns about PAEHRs, increased confusion, distress, or anxiety were anticipated by parents with no access [53]. Both parents with and without experience of PAEHRs worried about record access impairing the parents’ relationship with the provider [11,53] and, in turn, negatively affecting collaboration [53]. Another concern stemmed from empathy with HCPs, worrying that parental record access could increase the workload and lead to complications [51,53] or restrict communication between HCPs through the record [51].

### HCP Stakeholders

#### Overview

Comparatively fewer studies (11/74, 15%) explored HCPs’ experiences of or opinions on web-based access to EHRs for children, adolescents, and parents. Of these 11 studies, 8 (73%) were conducted in the United States, 2 (18%) were conducted in Australia [20,62], and 1 (9%) was conducted in the United Kingdom [37]. Most of these studies (6/11, 55%) were qualitative (focus groups or interviews), although the sample sizes were small; 18% (2/11) of the studies had a sample size of 1 [23,25]. In total, 9% (1/11) of the studies used a web-based survey [63], and 18% (2/11) used paper-based surveys [43,52]. Many studies (6/11, 55%) recruited representatives from a wide variety of clinicians, including, for example, specialist physicians, general practitioners, hospitalists, nurse practitioners, nurses, mental health clinicians, physician assistants, dietitians, physiotherapists, and pharmacists [20,37,43,53,62,63]. Survey studies ranged from 1 [23] to 212 [63] participants. Notably, only 18% (2/11) of the studies exclusively solicited the views of pediatric health professionals [62,64]. Several studies explored HCPs’ broad experiences with sharing PAEHRs with patients and parents [37,52,62]; a few focused on HCPs’ anticipation of the practice among children or adolescents and parents [25,43,53]. In total, 18% (2/11) of survey studies exclusively focused on providers’ perspectives on adolescent confidentiality with PAEHRs [63,64]. Only 12% (1/8) of the US studies reported on both accessibility and age of access: of 212 clinicians, 87.6% reported that their institution offered PAEHRs to both the adolescent and their parent or guardian, and most (69.1%) reported a minimum age requirement, with most (42.2%) citing between 12 and 14 years [63].

#### Positive Views and Experiences

Studies that explored HCPs’ experiences with PAEHRs among children or adolescent patients and parents reported positive experiences. For example, among 96 providers with experience sharing access at a children’s hospital, Kelly et al [52] found that 92% wanted patients and parents to continue to use the portal. They reported that patients and parents asked questions about the information they read, including laboratory test results (45%), medications (13%), and errors or mistakes in their care (3%). Exploring the views of HCPs in pediatric settings, Janssen et al [62] found that staff appreciated enhanced communication with patients, especially regarding coordinating appointments with parents and the potential for families or patients to ask questions. A study soliciting the views of 1 provider working in an adolescent inpatient psychiatric setting reported that clinical note sharing helped inpatient counseling sessions and compliance [23]. A study including 25 physicians identified experiences of increased transparency, improved documentation, reassurance or validation of concerns, and enhanced care plan clarity [39].

Among the anticipated benefits of sharing PAEHRs with child or adolescent patients and parents among HCPs with no experience of the practice, Kelly et al [53] reported that clinicians (including 8 nurses, 5 residents, and 7 hospitalists) predicted reinforced information, improved parental knowledge and empowerment, enhanced parent communication and partnership with providers, and increased provider accountability and documentation quality. Among 133 surveyed medical professionals, Chung et al [43] reported that 63.2% (84/133) believed that parental access may help identify incorrect information, and 61.7% (82/133) believed that access may reassure parents of the care provided to their child. In a qualitative study based in Australia by Beaton et al [20], school-based clinicians anticipated that adolescent patients with multiple providers would benefit from reduced duplication of investigations, ineffective treatment strategies, and more timely access to information.

#### Concerns and Negative Experiences

In several studies (4/11, 36%), patient confidentiality breaches and managing private patient information among children and adolescents was the leading concern [20,25,63,64]; as 1 surveyed clinician noted, “Privacy is just the biggest thing” [20]. In 18% (2/11) of the studies, HCPs reported that, despite sharing PAEHRs with other patients, they had precluded sharing information with adolescents because of privacy concerns, such as that savvy parents would be able to access it [20,25]; attesting to this, lack of clinician familiarity with PAEHR utility and technical implementation among minors was another expressed concern in both studies. Among clinicians with experience of PAEHRs, in a US study of 212 clinicians, nearly 4 in 10 (39.6%) were not at all confident that their EHR maintained privacy for minors, with 81.7% expressing concerns about maintaining confidentiality [63]. In another US study of 26 pediatric health care providers with experience of sharing PAEHRs with adolescents, Stablein et al [64] reported that confidentiality concerns affected documentation practices, such as worries that all HCPs involved in the child’s care will not be aware of what information in the record is private from parents versus what the parent needs to know, in addition to the fact that the record has a multifold purpose (eg, billing and communication with families). As a result, providers reported selectively omitting or concealing information and using codes on the EHR designed to alert other providers to confidential information.

Kelly et al [53] reported that HCPs with no experience of the practice (including 8 nurses, 5 residents, and 7 hospitalists) foresaw increased provider workload, heightened parental confusion, distress or anxiety, impaired parental relationship with providers, and compromised note quality and purpose. In a US study, 34% (17/50) of attending and intern physicians were concerned that parents would be confused by reading their child’s notes [39]. Among 133 surveyed medical professionals, Chung et al [43] reported that 114 (85.7%) believed that parental access may make medical professionals apprehensive about charting certain information, and 75 (56.4%) believed that parental access may increase the time spent updating parents, with approximately half (64/133, 48.1%) believing that parental access may increase the probability of a lawsuit. A study of inpatient pediatric physicians with experience of access found that 11% reported increased workload and 4% reported not being satisfied with portal use by patients or families [52].

### Other Stakeholders

#### Overview

The viewpoints of other stakeholders on pediatric PAEHRs constituted most of the included studies (30/74, 41%). These studies comprised three types of stakeholders: (1) experts (27/30, 90%) such as HCPs, IT experts, or researchers; (2) policy makers (4/30, 13%); and (3) the public (1/30, 3%). Of these 30 studies, 28 (93%) were conducted in the United States, 1 (3%) was conducted in Canada [21], and 1 (3%) was conducted in New Zealand [65]. The aim of many studies (15/30, 50%) was focused on ethical issues related to adolescent PAEHRs, and a few (2/30, 7%) described the development of a portal solution [66,67].

#### Positive Views and Experiences

Viewpoints focused mainly on concerns (which we describe in the following section) but included a number of positive views of PAEHRs for a pediatric population. Among informants from 25 medical organizations, it was stated that adolescent patients with chronic diseases benefited the most from parents having access [68]. In fact, pediatricians claimed that parents of children with chronic diseases should be offered full access to their children’s EHRs [69]. Jasik [70] advocated that PAEHRs could be useful in health education, in support of care transition for adolescents with chronic illnesses, and in risk behavior screening. Several viewpoints (3/30, 10%) argued that adding educational materials to the PAEHRs may facilitate literacy and comprehension for families [67,71,72]. Some noted unfulfilled potential for pediatric PAEHRs, for example, in the areas of patient data contribution [66], developmental screening [73], and research trial participation [74].

Green-Shook [75] anticipated that HCPs’ control of their schedule may increase with PAEHRs because of communication with patients via messaging rather than telephone, an anticipation that was subsequently observed in a primary care setting [48]. Several papers (4/11, 36%) reported a need for availability on mobile devices to increase accessibility and practicality for users [67,69,70,74], and a medical director developing a mobile PAEHR app advocated for the integration of various functions in one app [67].

#### Concerns

Most viewpoint papers included concerns about adolescent confidentiality [72,73,76-84]. HCPs in gynecology and psychiatry reported that adolescents may be less willing to seek health care if they are uncertain about confidentiality [82,83], and 83% of respondents in a public opinion survey [85] deemed adolescents less likely to discuss sensitive issues with HCPs when parents had access to their EHRs. An American organization advocating for adolescents’ health warned that adolescent aversion toward PAEHRs caused by confidentiality concerns and an uneven internet access could increase health disparities [86].

The studies described concerns in terms of portal functionality. Many insisted on an option for HCPs to label information as confidential [68,69,76,83,87] and enable adolescents to restrict parental access [80,86], some pointing to the variable definition of “sensitive” [68,81], which portal features contain such information [83], and division into “portions” of notes [84]. Psychiatric PAEHRs have been noted as unique in need of confidentiality, and Kendrick and Benson [83] listed portal functions that may hold information pertaining to sensitive topics in mental health, noting that sexual activity, gender identity, and substance abuse may be accessed in all portal areas. Bayer et al [80] posited that the release of sensitive information to the parent should require the adolescent’s consent, whereas Bourgeois et al [88] urged HCPs to carefully review notes to prevent leakage of sensitive information. Not only concerning children, 7% (2/30) of the studies noted the need for protecting caregiver privacy [73,76]. In fact, medical professionals favored customizable controls of information display for both parents and adolescents [69], and several studies prompted considering family circumstances [65,89]. A group of pediatricians suggested that structured data content could improve efficiency and consistency [73].

Jasik [70] asserted a lack of stakeholder investment in PAEHR development for adolescents and that current portals are usually not designed to deal with privacy issues. Attesting to this, pediatricians noted that adolescent access to patient portals is hindered by time-consuming decision-making and lacking technology and manpower and that implementation variability is a result of absent guidelines and vague laws [68]. Anoshiravani et al [69] proposed that portal access for adolescents should be limited until the privacy functionality is more robust.

Set age limits for patient and parental access to mitigate confidentiality issues has raised concerns and been the topic of much debate. Taylor et al [89] suggested different content access for three subgroups in the pediatric population (aged <13 years, 13-18 years, and >18 years) based on information sensitivity. Various studies (2/11, 18%) held that default ages may enable long-term consistency [65,68], allow for automated notifications, and facilitate policy making [65]. Conversely, viewpoint papers cautioned that age-based loss of access could seriously affect families reliant on EHR access in the care for a child [68]. With regard to the transition from child to adult, Sittig and Singh [78] discussed the transfer of EHRs created when the patient was a child, whereas Bourgeois et al [88] reported that their institution provided access “prospectively,” keeping confidential information suppressed also when the individual became an adult.

Several viewpoints (3/11, 27%) advocated for education on PAEHRs for various stakeholders [75,86,87], for example, that early HCP-initiated conversations with parents and adolescents may reduce parental concerns, increase acceptance [65], and set clear expectations [88]. Obstetrician-gynecologists have argued that adolescents should be informed if parents will have access to the EHRs [84], and Sherek and Gray [90] stated that, when possible, parents need to be informed of how to extend access to the child’s record. In a short paper, the American Academy of Child and Adolescent Psychiatry [91] provided advice for parents on questions for their child’s psychologist. In total, 13% (4/30) of the studies noted that insurance claims can lead to confidentiality issues [75,80,84,87], especially with uninformed use of the PAEHRs. The importance of guidance for staff has also been stated [69,88,92] as well as communication between staff and EHR vendors [68]. In pediatric psychiatry, Nielsen [81] advocated for training graduate students in penning PAEHRs. On the topic, a group of pediatric gastroenterologists recommended removing irrelevant details, not labeling emotions, and spell-checking [71].

Among other concerns, Gracy et al [73] described the divergent needs of pediatric portals compared with those of adult populations. Spooner [72] listed the critical areas for pediatric PAEHRs as immunizations, growth tracking, medication dosing, patient identification, norms for pediatric data, and privacy.

### Visual Summary of Stakeholders’ Expectations and Experiences

**Figure 4:**
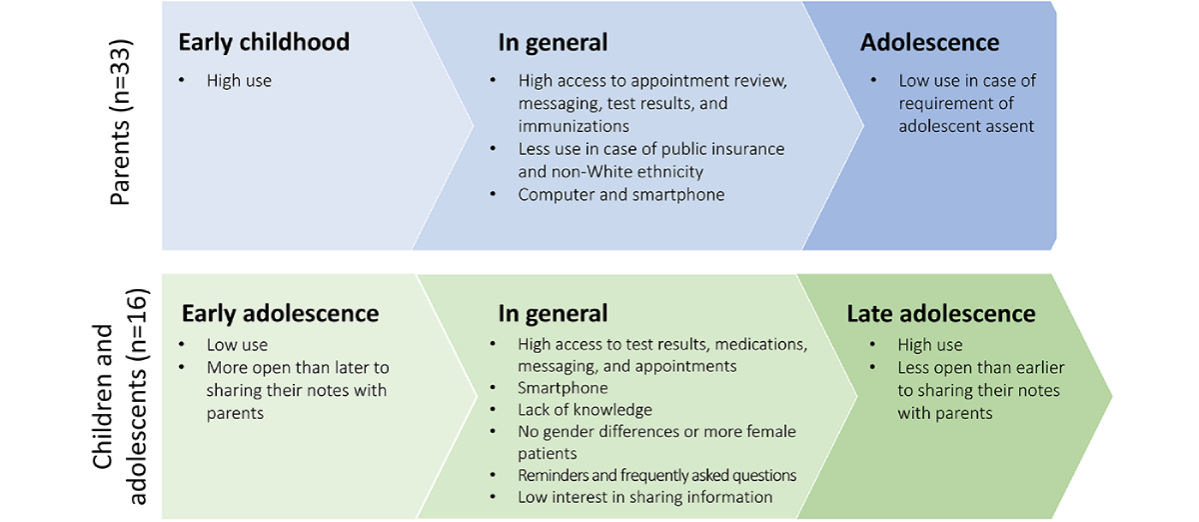
Summary of results of adoption and use of patient-accessible electronic health records among parents and adolescents. FAQ: frequently asked questions.

**Figure 5:**
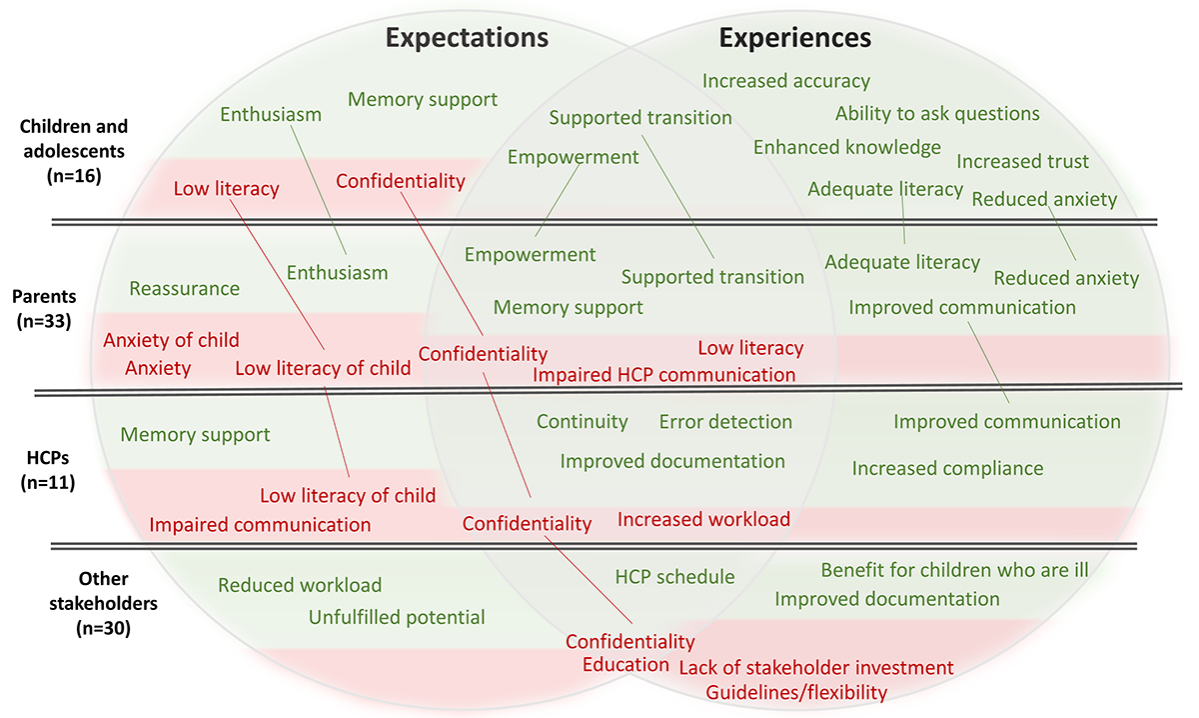
Summary of results of expectations and experiences of patient-accessible electronic health records among children/ and adolescents, parents, health care professionals (HCPs), and other stakeholders. Green text depicts positive views and experiences, and red text depicts negative views and experiences. Color in the various boxes illustrates the distribution of positive and negative views and experiences for the stakeholder group.

Figure 4 presents a visualization of the findings on adoption and use among adolescents and parents. Furthermore, Figure 5 provides a visualization of the findings based on expectations and experiences. Here, “expectations” is, as mentioned previously, a type of view in which the stakeholder has no previous experience of web-based record access.

## Discussion

### Principal Findings

The results of the 74 studies included in this scoping review contribute to the understanding of factors associated with stakeholders’ views, use, and experiences of children’s, adolescents’, and parents’ web-based access to the EHRs of children and adolescents. The reviewed studies consistently observed positive views and experiences on the part of parents and particularly of adolescents, whereas HCPs and other stakeholders held many concerns. In this section, we will (1) compare stakeholders’ views on and experiences with PAEHRs, (2) discuss some of the challenges that are unique to the PAEHRs of children and adolescents, (3) comment on the implications for design and implementation, and (4) suggest future research.

### Limitations

Although it followed the scoping review methodology, the review was limited by not assessing the quality of the included studies. By only including studies written in English, we may have missed important papers written in other languages. Considering that 92% (68/74) of the included studies were based in the United States, we do not know whether an information bias affected the findings. Among the identified studies, some merged adolescents with young adults or parent proxies, which complicated the analysis of specific groups. Furthermore, the studies’ definitions of adolescents varied, with the upper age limit ranging from 17 to 20 years. The studies did not always distinguish between positive and negative views or experiences. For example, the provision of education and guidance could be deemed as both a benefit and a concern. Furthermore, several expert viewpoints provided recommendations for the future based on concerns about PAEHRs, omitting to mention benefits. For the purpose of this study, we referred to the effects of PAEHRs that appeared beneficial to the patient as “benefits.” Finally, conducting stakeholder consultations after completing the review prevented any integration of their results into the study. Future scoping reviews may wish to invite stakeholders to a more active participation earlier or to provide input throughout the process.

### Expectations Versus Experiences Among Adolescents and HCPs

The findings suggest a similar pattern for adolescents to that previously observed in adult populations [9,10,93,97-99], where adolescents’ positive experiences contrast with HCPs’ concerns. For example, HCPs and parents imagined that adolescents would not understand the information in their notes and experience negative emotions as a result. However, adolescents reported high satisfaction and literacy even in the much-debated field of psychiatry. Another interesting aspect was that, although adolescents who had not previously accessed their EHR notes did have concerns about not understanding the notes and what parents may have access to, those with experience of accessing their records reported no such concerns.

A possible explanation for this might be a different perspective as most nonobservational studies exploring adolescents’ experiences with PAEHRs (4/5, 80%) included patients with serious illness or in inpatient settings. It might be that children and teenagers with serious illnesses may have a better understanding of medical jargon. In addition, they may be familiar with being dependent on parental insight into their care and involving parents in their health care issues. Thus, the adolescent’s desire for privacy is likely to depend on many factors, and there is still a need to provide confidentiality for those who require it, which was mentioned in many viewpoint papers.

The one existing review in the field [14] did not include expectations of PAEHRs; however, its findings in terms of experiences were aligned with our included evidence. For example, there was enthusiasm among adolescents and interest among parents in using patient portals, whereas medical literacy and confidentiality were the main concerns. Similarities are not surprising as, of the 11 included papers in the aforementioned review, only 3 were not included in this review (because they did not have a focus on pediatric PAEHRs). Except for not focusing on expectations, among the differences were that the previous review included use barriers and clinical outcomes and did not include the perspectives of HCPs and other stakeholders.

Interestingly, all but one of the parents’ concerns about adolescents’ confidentiality referred to external parties rather than the self as a parent as a threat to their adolescent’s privacy. It is difficult to explain this result, but it might be related to the fact that parents have been found to value the importance of their involvement highly out of concerns about not being apprised of important information and uncertainty of the child’s ability to manage their own care [100]. Instructing HCPs to engage parents and adolescents in a dialogue on confidentiality has been mentioned previously as a strategy to mitigate parents’ worries, although current extensive pressure on HCPs may necessitate new approaches to such education.

### Special Challenges for Pediatrics

A key challenge for PAEHRs is balancing confidentiality and information privacy for adolescent patients with the need for parental involvement in the adolescent’s care. Several viewpoint papers focused on guidelines regarding when and how to grant access to parents and adolescents. The results are inconclusive and reflect the complexity of this issue. A health institute argued that allowing for manual changes to parental access can signal that the child has received some type of sensitive care [27]. Set age limits for automatic gain and loss of access could be beneficial, yet an extensive variety of potential circumstances do call for customizability according to the situation. A lack of investment and priority of portal development for adolescents and parents was indicated, which one could argue causes a waste of potential of PAEHRs and a loss for the health care system in the long term. One such function advocated by numerous viewpoints was the possibility of designating information as confidential. Still, efforts to hide sensitive information from parental view could be counteracted by parents evading the system to access their adolescents’ accounts directly. If a parent perceives their adolescent incapable or unwilling to manage their own health care, they may consider it necessary and part of their parental responsibility to find a work-around. A recent UK article published outside the search period indicated that more than half of the messages to adolescents’ accounts were accessed by guardians [101]. In addition to protecting the adolescent, a few papers stressed the importance of considering caregiver privacy in cases where parents disclose confidential information with regard to the child’s care. Furthermore, modern family constellations vary, which may require the consideration of access provision based on the type of parental or legal guardianship. In a case study, health data coordinators at a US medical center described using different rules of access for a “natural or adoptive parent,” legal guardian, or stepparent [90]. The same institution denied parents aged <18 years access to their child’s EHR before becoming an adult, highlighting another potential issue. Differences between countries further complicate the issue of PAEHRs in pediatrics; for example, the definition of policy maker in the PAEHR context varies considerably by country, whereby HCPs in some countries are required to decide on policies themselves.

### Consultation With Stakeholder Representatives in Sweden

We consulted on the findings with a pediatric oncologist, a young patient council at a public hospital in Sweden, and the Ombudsman for Children in Sweden. All reported their feedback via email. First, the pediatric oncologist reported not missing any aspect in the results. She reported that she considered the findings highly interesting and the biggest takeaway to be the positive effects on adolescents and parents of reading the PAEHRs and that security seemed to be the main cause of worry. Second, the young patient council reported to the first author, after discussion in a meeting, that the findings “looked very good” and dovetailed with their own experiences of having access to the EHRs. They reported that they had nothing to add to our findings. Third, the Ombudsman for Children in Sweden expressed positivity toward this overview as none has so far been done. He had questions about the findings, such as about results that confirmed his suspicions (eg, that male patients were more likely than female patients to consider allowing proxy access), as well as whether there was a complete lack of Swedish studies. He also asked for clarification of one case of unclear wording. An area that he saw as missing was the perceptions of shared access among adolescents and parents. As a result, we clarified some wordings and included the perspective of shared access in the Results and Discussion sections.

### Implications of the Findings

Consistent findings can be summarized into four implications for PAEHR implementation: (1) adolescents and parents should be educated on PAEHR use and confidentiality (eg, information visibility for children, adolescents, and parents; possibility to restrict information; reasons for age limits; children’s and adolescents’ need for privacy; the moment when parents will lose access; and procedures for parents to stay involved in the child’s care); (2) HCPs should communicate with EHR vendors and be educated on PAEHRs (eg, use; updates; privacy functionality; and information visibility for children, adolescents, and parents); (3) PAEHRs should be available on mobile devices, and functions need to be integrated; and (4) there should be options on a portal for HCPs and patients to label information as confidential.

### Future Research

There is a lack of studies examining the effects of PAEHRs among children and adolescents. Although the Nordic countries are often considered to be at the forefront of PAEHR implementation [1] and access has been available longer at the national level in Sweden than in most other countries, no survey studies targeting a pediatric population in Sweden have been published to date. However, there is ongoing research within the NORDeHEALTH project [1] (with some of the authors’ involvement) that aims to rectify this situation. One way is to explore approaches that have already been implemented and conduct comparative studies on the benefits and risks of access or exclusions among children and adolescents. Owing to the current scarcity, investigations with focus on literacy and confidentiality in adolescent outpatient or nonclinical populations are suggested. In addition, there is a need to explore the anticipation of parents and adolescents that shared access may support the transition to adulthood. Furthermore, there is little evidence on the efficiency of PAEHRs in the pediatric population, and work should be undertaken to better understand the effects on documentation time for HCPs and the potential cost-effectiveness of PAEHRs for families and adolescents in the long term. Finally, questions remain with respect to how PAEHRs affect the quality of documentation [102]. In this area, the approach of natural language processing has been increasingly used to quantitatively examine note changes, for example, according to ethnicity and disease chronicity [103].

### Conclusions

This study consisted of a scoping review of 74 studies on PAEHRs for parents, children, and adolescents. Most studies (27/74, 36%) were comment papers as, despite the urgency of the matter, there is limited research, particularly regarding adolescents’ experiences with web-based access to their records and outside the United States. Existing literature on pediatric PAEHRs indicates a pattern similar to that observed in adult populations, whereby adolescents’ and parents’ strong interest and positive experiences of accessing the records are juxtaposed with and obstructed by concerns among HCPs and other stakeholders, confidentiality being the key issue. Our findings could inform the design and implementation of future regulations regarding access to PAEHRs. Further examination of the experiences of adolescents, parents, and HCPs is warranted to improve usability and utility, inform universal principles reducing the current arbitrariness in the child’s age for own and parental access to EHRs among providers worldwide, and ensure that portals are equipped to safely and appropriately manage a wide variety of patient circumstances.

## Appendix

Multimedia Appendix 1 PRISMA-ScR (Preferred Reporting Items for Systematic Reviews and Meta-Analyses extension for Scoping Reviews) checklist.

Multimedia Appendix 2 Summary of the included studies.

## Abbreviations

EHR: electronic health record
HCP: health care professional
PAEHR: patient-accessible electronic health record
PRISMA: Preferred Reporting Items for Systematic Reviews and Meta-Analyses
PRISMA-ScR: Preferred Reporting Items for Systematic Reviews and Meta-Analyses extension for Scoping Reviews
