# Supplementary material for: Views, Use, and Experiences of Web-Based Access to Pediatric Electronic Health Records for Children, Adolescents, and Parents: Scoping Review
Source: J Med Internet Res. 2022 Nov 22;24(11):e40328. doi: 10.2196/40328 (PMC9727693; doi:10.2196/40328)
Supplement: Multimedia Appendix 2 [file jmir_v24i11e40328_app2.pdf]

Multimedia Appendix 2. Summary of included studies

Some studies were made before implementation of online record access, and capture different stakeholders' expectations, whereas others study the actual experiences of accessing the record online. In this table we have marked (where relevant) this with (experiences) or (expectations) in the column Purpose.

Authors	Year	Ref-ID	Country	Study design	n	Participants	Setting	Purpose	Results
Ahlers-Schmidt et al.	2013	[45]	USA	quantitative	171	parents	Pediatric (expectations and experiences)	To describe parent feedback and intention to use the PAEHR related to their children's EHR after a facilitated learning session.	Most parents had no prior experience with the patient portal but were satisfied overall with ease of use and PAEHR features. HCPs should consider (1) hands-on demonstrations of the patient portal to encourage understanding and use and (2) alternative access venues if home computers are not available.
Ancker et al.	2018	[89]	USA	quantitative	N/A	other stakeholders (public opinion)	non-clinical (expectations)	To explore the public opinion about adolescent medical privacy.	Public opinion was in favor of parental access. A brief "nudge" increased acceptance of adolescent confidentiality. Public believed parental access will lead adolescents to withhold health information from HCPs.
Anoshiravani et al.	2012	[73]	USA	comment	N/A	other stakeholders (IT experts)	N/A<br>adolescent care	N/A	Authors advocate a number of PAEHR functionalities for adolescent patients, including default privacy settings, customizable privacy controls, and proxy access capabilities when necessary.
Arnott Smith et al.	2020	[54]	USA	qualitative	8	parents	pediatric inpatient (expectations)	To identify parent perspectives of HCPs sharing notes with parents during the child's hospitalization.	Multiple potential benefits of and challenges were anticipated. Benefits included memory reinforcement, improved knowledge, improved communication, ensured continuity of information, and improved empowerment. Concerns included note content, impaired communication with and between HCPs, and impact on family or patient.
Asan et al.	2019	[59]	USA	qualitative	33	parents	pediatric inpatient intensive care (experiences)	To explore perceptions of parents of pediatric ICU patients of real-time EHR displayed in patient rooms.	Access to open electronic health record data may be an effective way to empower and engage parents in the PICU, but potential drawbacks were also noted.
Bayer et al.	2015	[84]	USA	comment	N/A	other stakeholders (researchers)	N/A<br>adolescent care	N/A	There are ethical, technical, political and economic issues to adolescent confidentiality, in addition to a previously unmentioned need to protect the parent's health information in adolescent's EHR from the adolescent. Sensitive data should require consent, yet there are exceptions when information release of information is ethically justified.

Multimedia Appendix 2. Summary of included studies

Beaton et al.	2021	[23]	Australia	qualitative	adolescents n = 8<br>HCPs n = 18	adolescents (age 15-16) and HCPs	non-clinical (school) (expectations)	To examine adolescents' understanding of the national PAEHR, and school-based HCPs' knowledge and their use of the PAEHR with adolescents.	Adolescents: Low understanding and use of the PAEHR, but satisfaction with level of control was reported after receiving a demonstration. Some wanted sensitive information shown to reduce awkwardness. Wanted to selectively restrict access, identify who had accessed their EHR and remove access to any person. Could be memory aid.<br>HCPs: concerned with adolescents' privacy and confidentiality, felt a need for education.
Bell et al.	2021	[48]	USA	quantitative	3333	parents	outpatient, most medical and surgical specialties (experiences)	To examine the effect of reading notes on family perceptions of safety-related knowledge and behaviors.	Parents who read visit notes report greater adherence with tests, referrals and medications, feel more informed and more likely to check results.
Bergman et al.	2008	[27]	USA	qualitative	adolescents n = 35<br>parents n = 34	adolescents (age 16-18) and parents	outpatient (expectations)	To assess parents' and teens' attitudes toward the use of a patient portal.	Adolescents: Enthusiasm, PAEHRs considered a step towards own involvement of their own healthcare. Appreciated the convenience of booking appointments online and secure messaging. Wanted: to receive information in the future, to be invited to sign up, to be able to sign up without parents' approval, and choose the features they want to use. Concerns included confidentiality and literacy, wanted parents to pay without seeing details.<br>Parents: Enthusiasm and desire to learn about the PAEHR from HCPs. Recommended an orientation session for teens. Differing views regarding billing information were expressed. Concerns included lack of awareness about appointment booking and of important information around the sexual health, and the adolescent not being able to process the information. Wanted to have to consent for their teens to use PAEHRs and for feature availability. Distrust was reported in that their teen knowing what the parent needs to know.
Berlan et al.	2009	[81]	USA	comment	N/A	other stakeholders (HCPs)	adolescent care	To review healthcare rationale and legal framework for confidential care to adolescents.	Ease of EHR access is a future challenge for adolescent confidentiality. Parents are often conflicted in their views of confidential care, however are often receptive to changing their views. Existing legal framework supports

									provision of confidential care, new tech may allow inadvertent privacy breaches.
Bialostozky et al.	2020	[70]	USA	quantitative	N/A	other stakeholders (HCPs)	N/A Pediatric outpatient subspecialty (experiences)	To describe their journey of sharing notes.	Default release of notes in pediatric organizations to led to more sharing of notes than requiring HCPs to choose to share notes.
Bourgeois et al.	2018	[92]	USA	comment	N/A	other stakeholders (HCPs)	N/A Pediatric and adult (experiences)	To discuss three illustrative cases highlighting common pediatric OpenNotes concerns.	Guidance for organizations and HCPs regarding documentation practices and PAEHR policies, to promote patient engagement and information transparency while upholding patient and parent confidentiality and the patient- and/or parent-HCP relationship.
Bourgeois et al.	2019	[29]	USA	quantitative	Not specified (398 reports)	adolescents (age 13-17) and parents	outpatient and subspecialty visit notes including medicine, surgery, adult care and pediatrics (experiences)	To explore patient and family EHR error reports.	Common types of reported inaccuracies included inaccurate description of symptoms or reasons for the visit (21%), other health problems (21%), and medication list (18%), missing information (15%). Errors were both minor and major. Relatively few reported bothersome language.
Bourgeois et al.	2008	[80]	USA	comment	N/A	other stakeholders (HCPs and policymakers)	N/A Pediatric and adolescent	N/A	Framework for understanding and addressing the challenges posed by child, adolescent, and family access to PAEHRs.
Bourgeois et al.	2018	[57]	USA	report/comment	N/A	other stakeholders (HCPs)	N/A Pediatric and adolescent (experiences)	To explore how patients and their doctors in pediatrics perceive having shared access to notes.	Enthusiasm and support for record access. Reported benefits for parents include memory aid for overwhelmed parent, availability when only one parent is allowed to attend. Benefits for adolescents include education and empowerment, self-care and self-advocacy. Challenges: adolescents sometimes able to seek medical care without parental consent, and information from such private visits must not be shared with parents if adolescents wish to not disclose it. Furthermore, parents may disclose information they wish to not disclose to the adolescent or the other parent. HCPs must be careful to protect information and respect either party's wish for privacy. Benefits to shared access: 1) enabling patients and/or parents to better absorb information shared by healthcare providers; 2) assisting a parent who cannot attend a healthcare visit in gaining a better understanding of what was

									learned; and 3) supporting adolescent empowerment related to healthcare needs.
Britto et al.	2013	[11]	USA	qualitative	24	parents	pediatric inpatient and outpatient, chronic illnesses: cystic fibrosis, juvenile idiopathic arthritis, or diabetes mellitus (experiences)	To examine parents' perceptions of the benefits and/or drawbacks of a PAEHR for managing their child's chronic illness.	PAEHRs have numerous user-perceived benefits for the management of chronic illness.
Britto et al.	2009	[41]	USA	mixed-methods	16	parents	pediatric inpatient and outpatient, chronic illnesses: cystic fibrosis, diabetes or arthritis (experiences)	To evaluate the usability of PAEHRs for parents.	Some difficulties observed for the parents, thus usability testing can be helpful in making healthcare system interfaces for laypersons more user-friendly and potentially more functional for patients and their families
Burke et al.	2010	[52]	USA	quantitative	Not specified (270 families)	parents	pediatric cardiology (experiences)	To explore how families utilize a web-based multimedia PAEHR, for patients of children with congenital cardiac disease.	Adoption rate was 93%. Users accessed the PAEHRs more whilst in hospital than after discharge. There were more views of imaging data than of textual data. The average log-in number was 25 (range: 1-440). Of 12 patients who died during study period, 11 of their families continued to access the records afterwards. Two families accessed the records more than 1 year after the child's death. paper charts during the study period.
Byczkowski et al.	2014	[60]	USA	mixed-methods	126	parents	pediatric outpatient: cystic fibrosis, diabetes mellitus, or juvenile idiopathic arthritis (experiences)	To understand perceptions of the usability and value of web-based PAEHRs among parents of children with a chronic condition.	Parents agreed that PAEHRs improved (1) their ability to manage and understand their child's condition (70%), (2) their communication with HCPs (62%), (3) their relation with their HCP, and (4) decision-making for their child. Parents appreciated not having to rely on HCPs for information (56%).
Byczkowski et al.	2011	[50]	USA	quantitative	Not specified (1900 families)	parents	Pediatric inpatient and outpatient: diabetes mellitus, juvenile idiopathic arthritis, or cystic fibrosis (experiences)	To assess the use of PAEHRs among families of children with chronic diseases and to describe characteristics of portal registrants and users.	Understanding the feasibility of portal use by parents is an important first step to using portals for improving self-management, patient-provider interactions, and outcomes for children with chronic diseases. Differences in adoption identified!

Calman et al.	2014	[30]	USA	quantitative	adolescents n = 1534<br>parents n = 97	adolescents (age 10-18) and parents	outpatient (experiences)	To describe a process for implementation policies for adolescents, and explore how we can balance adolescent confidentiality with the need to involve parents in adolescents' care.	Overall, 11 percent of patients ages 10 to 17 activated a portal, compared with 31 percent of the general patient population. Most frequently used features for adolescents were messaging, appointment scheduling, and review of lab test results. There were no complaints from either parents or adolescents about confidentiality.
Carlson et al.	2020	[28]	USA	qualitative	parent n = 1<br>adolescent n = 1<br>HCP n = 1	adolescent (high school senior), parent, and HCP	hematology (experiences)	To explore what different stakeholders think about PAEHRs and adolescents' needs of confidentiality.	Adolescent had low initial knowledge about PAEHR use. Difficult for child to manage everything about their care, however need increases as one becomes more independent and goes to college. Parent-teen relationship may affect teen's perception of portal. There should be a possibility to make e-mails private in the messaging system. A mobile append and text reminders were recommended.
Chung et al.	2018	[46]	USA	quantitative	parents n = 85<br>HCPs n = 133	parents and HCPs	inpatient neonatal intensive care (expectations)	To explore parent and HCPs' opinions regarding independent parent access to their child's EHR during NICU hospitalization.	The spirit of the American Academy of Pediatrics (AAP) recommendation supporting family-centered care can-not be realized without some degree of independent parental access to their child's EHR.
Dalrymple et al.	2018	[44]	USA	quantitative	270	parents	pediatric (expectations)	To learn about patients' and families' point of view during implementation of a PAEHR.	Patients' parents were eager to use PAEHRs to manage their child's care.
Dohil et al.	2021	[26]	USA	quantitative	adolescents n = 20<br>HCP n = 1	adolescents (age 12≤) and HCP	inpatient psychiatry (experiences)	To evaluate whether AYA patients hospitalized at our inpatient psychiatric ward might similarly benefit from note sharing.	AYA patients with active behavioral health concerns understand and express general satisfaction with their medical documentation.
Edwards et al.	2020	[64]	USA	quantitative	96	parents	pediatric radiology (expectations)	To determine how the parents of pediatric radiology patients prefer to receive the results of their child's imaging studies.	Pediatric radiology patients prioritize receiving radiology results quickly, but only six percent preferred to receive results via PAEHRs. Most still prefer to receive results by their child's doctor in-person (37%) or by phone or e-mail (28%), by a radiologist in-person (16%), via a printed report copy (7%) or mail (3%).
Frazer et al.	2017	[24]	Canada	mixed-methods	survey = 1006<br>6 focus groups	adolescents (age 12-19)	non-clinical (society) (expectations)	To research how Canada provincial and territorial privacy laws apply for minors and "mature minors" and their right to access their EHRs.	Both adults and adolescents agree personal health information is confidential is right of adolescent to view electronically.

Gaskin et al.	2016	[65]	USA	qualitative	83	parents	non-clinical (juvenile detention center) (expectations)	To examine parental attitudes towards the release of health information to adolescents.	Parents appear supportive of allowing their adolescent children to have online access to and full control of their own personal health information. Parents' primary concerns are related to privacy and security features.
Goldstein et al.	2020	[67]	USA	quantitative	212	HCPs	N/A<br>Adolescent care (experiences)	To explore clinicians' perspectives on EHR functionality and institutional policies related to confidentiality and health information sharing for adolescents aged <18 years.	Despite high comfort levels and robust PAEHR functionality at many institutions, significant concerns about adolescent confidentiality remain. Varying institutional approaches to protecting confidentiality underscores the need for a standardized and comprehensive framework to enable providers and institutions to take better care of adolescents.
Gracy et al.	2012	[77]	USA	comment	N/A	other stakeholders (HCPs)	pediatrics	To discuss historical reasons, review special requirements for PAEHR design to meet users' needs, provide sample cases and offer recommendations and considerations.	PAEHRs provide significant opportunities to improve pediatric care. Well-constructed clinical content, HIE, automated reminders and alerts, and reporting at practice, community and public health levels are available in several current systems and products.
Gray et al.	2014	[90]	USA	comment	N/A	other stakeholders (HCPs)	N/A<br>Adolescent care	N/A	As adolescence can benefit from access to EHR, some issues need to be considered before. Weak points are addressed and recommendations made.
Green-Shook	2009	[79]	USA	comment	N/A	other stakeholders (researchers)	N/A<br>Health info man.	To explore issues about releasing children medical notes to parents.	Proxy access requests will grow and millennials will look at it as expectation, not as option. Sharing medical notes is tricky and need some legislation improvements.
Hong et al.	2016	[38]	USA	mixed-methods	adolescents, n = 23<br>parents, n = 23	adolescents (age 13-17) and parents	oncology, hematology (cancer, blood disorders) (experiences)	To investigate experiences with MyChart, a tethered PHR system.	Adolescents: Quick access to lab results was found to ease adolescents' frustration about delays. Reflected more on their illness event, and information led them to ask questions that they might not have known to ask before, and had slightly higher expectations that the PAEHR would enable them to improve their health. Relied on parents to explain information that was unclear to them, or seeking out information elsewhere online that was not readily available on the portal. No confidentiality concerns. Preferred smartphones or tablet devices for using PAEHRs.

									Parents: More likely than the patients to find it difficult to track their child's health and desire for a different Portal design, and to use the PAEHR to communicate with HCPs. Concerned about negative results only visible to the adolescent.<br>Both groups saw value in using the portal, and reported reading the PAEHRs in advance could help when talking to their doctor. The PAEHR was used to review notes and check the information was correct. Both groups agreed that the portal could serve to support the transition from pediatric to adult care, and better communication with HCPs.
Huang et al.	2019	[35]	USA	quantitative	55	adolescents (age 12-17)	chronic disease Gastroenterology, hepatology (experiences)	To examine results of medical note sharing among adolescents with chronic disease.	Adolescents reported high satisfaction and adequate comprehension with their medical documentation at medical visits.
Janssen et al.	2021	[66]	Australia	qualitative	11	HCPs	pediatrics (experiences)	To understand healthcare teams' experiences of using a patient portal and explore the barriers and enablers to ongoing use	Value for the patient, improved workflow, and adequate technical and implementation support were highlighted by participants.
Jasik	2016	[74]	USA	comment	N/A	other stakeholders (HCPs)	N/A<br>Adolescent (experiences)	To present a detailed account of their institutional process for a patient portal and its approach to adolescents and parents.	A major barrier to PAEHR implementation for adolescents and parents is lack of stakeholder investment.
Kelly et al.	2020	[55]	USA	mixed-methods	47	HCPs	pediatric inpatient (experiences)	To evaluate provider experiences with a bedside PAEHR application for hospitalized patients and families in a children's hospital.	Patients/parents asked providers questions about information in the portal, pertaining to test results, medication, diagnosis and errors found. 92% (all but 1) of providers wished for continued portal access for hospitalized patients and families.
Kelly et al.	2017	[61]	USA	mixed-methods	296	parents	pediatric inpatient (experiences)	To assess portal use and parent experiences and perceptions of the impact of portal use on inpatient care.	Parents were satisfied with the PAEHR. Portals may engage parents in hospital care, facilitate parent recognition of medication errors, and improve perceptions of safety and quality.
Kelly et al.	2021	[56]	USA	qualitative	parents, n = 8<br>HCPs, n = 26<br>(nurses 8, residents 5, hospitalists 7, administrators 6)	parents and HCPs	pediatric inpatient (expectations)	To identify the perspectives of parents, HCPs, and hospital administrators on the anticipated benefits and challenges of giving parents access to HCPs' notes during hospitalization, and	Parents anticipated e.g. improved knowledge, increased empowerment, and enhanced communication and trust.<br>HCPs and administrators anticipated improved accountability and documentation quality, but were concerned about effects on workload.

								strategies on how to implement the note sharing process.	All groups anticipated benefits including reinforcement of information for parents. All groups had concerns about 'bad news' being shared via the PAEHRs and confusion due to not understanding medical jargon.
Kelly et al.	2019	[58]	USA	qualitative	14	parents	pediatric inpatient (experiences)	To (1) identify why parents used an inpatient portal application on a tablet computer during their child's hospitalization and 2) identify their perspectives of ways to optimize the technology.	Parents used the PAEHR to track their child's progress, to feel empowered and rely less on HCPs, because it facilitated communication, to ensure information accuracy, and as memory aid.
Kendrick et al.	2017	[87]	USA	comment	N/A	other stakeholders (HCPs)	pediatric psychiatry	To discuss the concept and implementation of patient portals in the child and adolescent population with a special focus on psychiatry.	The use of PAEHRs in pediatric psychiatry could offer numerous benefits to HCPs and patients, and a new way to engage patients in their care. Confidentiality issues will complicate portal use with this population but with well-considered policies, sharing should be possible.
Ketterer et al.	2013	[51]	USA	quantitative	84015	parents	pediatric outpatient (experiences)	To identify the demographic, practice site, and clinical predictors of patient portal enrollment and activation among a pediatric primary care population, and to describe use of the portal.	A number of sociodemographic disparities were identified: Predictors increasing the odds of portal activation included higher income, and lower age child group, higher visit count, longer problem list, longer distance from clinic.
Knopf	2021	[95]	USA	comment	N/A	other stakeholders (HCPs)	N/A Adolescent psychiatry	To provide guidance to guardians on how to advocate for their child.	The author provided tools and information from the American Academy of Child and Adolescent Psychiatry for parents on working with psychiatrists, for example, advice on questions about confidentiality, patient portals, and health records.
Krasowski et al.	2017	[31]	USA	quantitative	Not specified (59388 unique patients)	adolescents (age 12-17) and parents	inpatient, outpatient, intensive care, emergency (experiences)	To describe a retrospective analysis of patient access to diagnostic test results released through PAEHRs.	Adolescent adoption was low. The highest rates of patient portal activation were for females 11 years or younger and 26–50-year-old. Males between the ages of 12 and 17 years had the lowest percentage of viewing test results even though approximately 40% of patients in this age range had an active patient portal account.
Lam et al.	2021	[43]	USA	mixed-methods	3339	parents	outpatient (experiences)	To describe attitudes, experiences, and barriers related to reporting perceived serious note errors.	Among study participants, nearly half of them found errors in their PAEHRs and did not report. About 1/5 of participants recommended new reporting tools that do not yet exist.

Multimedia Appendix 2. Summary of included studies

Lee et al.	2021	[75]	USA	comment	N/A	other stakeholders (HCPs)	pediatric gastroenterology	To raise awareness of how adapt medical documentation according to 21CCA.	Authors stated that patient access to their medical records will have a great impact in the clinical practice of pediatric gastroenterology. For example, educational materials can be added to information release. Practical recommendations for writing notes were provided.
Miklin et al.	2019	[37]	USA	quantitative	97	adolescents (age 13-18)	outpatient (expectations)	To assess barriers to PAEHRs in a culturally diverse adolescent population.	94% of patients had heard of the term EHR but only 55% were familiar with its function. HCPs should be active in educating adolescents about EHR portals, including with respect to sensitive and confidential medical concerns. In total, 84% of participants expressed interest in viewing their records online and reported a mean score of 8.46 out of 10 when asked if they would like to see the test results online
Nielsen	2015	[85]	USA	comment	N/A	other stakeholders (HCPs)	psychiatry	To highlight the ethical considerations that arise with use of PAEHRs in pediatric settings.	The author stated that due to risks to confidentiality with PAEHRs of adolescents, HCPs must take caution when documenting information that adolescents do not want shared with parents or guardians.
Park et al.	2015	[78]	USA	comment	N/A	other stakeholders (HCPs)	N/A<br>pediatric	To discuss adaption of PAEHR functionality for pediatric patients.	PAEHRs hold potential to help adolescents to manage their health, but attention must be paid to privacy and confidentiality considerations. Proxy options will be helpful to ensure privacy.
Ramsey et al.	2018	[36]	USA	quantitative	96	adolescents (age 13-17.9)	academic outpatient (experiences)	To examine feasibility, acceptability and effectiveness of intervention to increase PAEHR sign-up, as well as examine satisfaction of a PAEHR app. primarily serving low-income urban African American patients and families.	Adolescents were either satisfied or very satisfied with the PAEHR and had an active interest in using PAEHRs. males were more likely than females to consider allowing proxy access
Ransom	2016	[25]	USA	comment	N/A	adolescent (age 15)	N/A<br>Non-clinical	To advocate the rights and needs of teenagers to access their EHRs.	There is a need for adolescents to access their notes: as memory aid, to learn how to take care of their own health, to prepare for adulthood. There are exceptions where access may not be beneficial, such as psychiatric notes. Being trusted will render adolescents more responsible and mature.

Ronis et al.	2015	[53]	USA	quantitative	184	parents	outpatient (expectations and experiences)	To assess acceptance, barriers, and intentions regarding use of PAEHR for their children with ADHD.	Parents are enthusiastic about PAEHRs. Future work should focus on engaging them as members of the health care team
Sarabu et al.	2021	[62]	USA	quantitative	159	parents	pediatric outpatient (experiences)	To better understand how pediatric patients and families perceived OpenNotes.	PAEHR was well-received by parents of pediatric patients without untoward consequences. Main concerns of HCPs proved to not be issues in the pediatric population.
Schapiro et al.	2021	[96]	USA	comment	N/A	other stakeholders (policymakers)	N/A adolescent	To review implications of the open notes requirement of the Cures Act, suggests strategies to improve care for adolescent patients, and recommends advocacy to improve the 2020 Final Rule implementation.	Authors stated that the Cures Act does not address the implications of open notes for confidential adolescent care. HCPs should work with information technology experts to ensure options are available for separating confidential information for adolescent patients, HCPs should be trained about the Cures Act, PAEHR providers should expand capacity to block information, and HCPs should advocate for exceptions to open notes at the federal level.
Schneider et al.	2016	[40]	UK	qualitative	parents n = 3<br>HCPs n = 3	parents and HCPs	pediatric inpatient, chronic illness (experiences)	To investigate patient families' lived experiences of working with PAEHRs.	How families coped with their child's chronic condition was strongly related to their PAEHR use. Not all families are willing to take more control and responsibility for their health management, and are differently motivated to use PAEHRs. PAEHR should be designed to meet the needs of avoidance-oriented users, to help activate their engagement and usage.
Sharko et al.	2018	[72]	USA	qualitative	Not specified (informants from 25 medical organizations)	other stakeholders (policymakers)	N/A adolescent	To determine the diversity in adolescent PAEHR policies across a range of institutions and determine the factors influencing decisions about these policies.	Adolescent PAEHR policies are highly inconsistent across the US. Medical organizations implemented different degrees of adolescent access, degrees of parental access, and types of information considered sensitive. Absent universally accepted standards, medical organizations weigh local legal, economic, social, clinical and technological factors in deciding access.
Sherek et al.	2014	[94]	USA	comment	N/A	other stakeholders (IT experts)	N/A pediatric	To describe and reflect on the management of PAEHRs in pediatric population for one health institution.	Authors stated that granting portal access requires reflecting on state laws on minors' rights. Policies and procedures on portal access rights, however, will continue to evolve. Organizations must consider complex

									family relationships, privacy and security standards, and legal and regulatory standards.
Sittig et al.	2011	[82]	USA	comment	N/A	other stakeholders (researchers)	N/A<br>Adolescent	To describe and discuss legal, ethical, and financial questions threaten the widespread adoption and use of PAEHRs.	Adolescent control of access to their data was reported as an ethical dilemma, stating that HCPs should aim to maintain adolescents' rights. Another issue is the transfer control of PAEHRs created when the patient was a child.
Spooner	2007	[76]	USA	comment	N/A	other stakeholders (HCPs)	pediatrics	To review functions of importance to child health care providers for electronic health systems.	There are some functional areas that are so critical to the care of infants, children, and adolescents that their absence results in the system impeding quality pediatric care.
Stablein et al.	2018	[68]	USA	qualitative	26	HCPs	outpatient clinic and inpatient service	To understand pediatric healthcare providers' expectations and practices in protecting patient confidentiality with PAEHRs. (experiences)	PAEHRs present new demands, including new challenges for maintaining confidentiality. PAEHR design and development will be fundamental to protect adolescent privacy.
Steitz et al.	2017	[32]	USA	quantitative	adolescent users (including proxy users), n = 2720<br>parents, n = 16036	adolescents (age 14-17.9) and parents	pediatrics (experiences)	To explore how pediatric patients and their caregivers use PAEHRs.	Pediatric patients and caregivers have avidly used messaging, test result, and appointment functions. The majority of access was done by surrogates. Adolescent portal usage increased with age. Most accounts for pediatric patients were only used actively for a few years, with peak usage for patients in early childhood and late adolescence.
Swartz	2013	[83]	USA	comment	N/A	other stakeholders (researchers)	N/A<br>adolescent	To address a policy statement.	A summary of a recent policy statement and the background of the issue was provided. Authors contended the importance of protect the rights of adolescents to consent for care and information release.
Taylor et al.	2015	[86]	USA	comment	N/A	other stakeholders (HCPs)	N/A<br>Pediatric and adolescent gynecology	To discuss PAEHRs and confidentiality in the context of adolescent care.	Authors held that HCPs must familiarize themselves with the laws concerning confidential services and with the specific capabilities of their PAEHRs to protect patient privacy, and educate their adolescent patients and their families about confidentiality. HCPs must also engage EHR vendors and IT professionals to ensure that adolescent privacy needs are met.
Taylor et al.	2007	[93]	USA	comment	N/A	other stakeholders (researchers)	N/A<br>pediatrics	To identify medical information that needs user-specific access controls for a pediatric PAEHR and identify areas in which access policies would be required to	Three subpopulations (<13, 13-18, 18<) in pediatric PAEHRs were identified. Access control policies need to handle developmental and age-defined rights of users, and to preserve privacy. This requires rules

								govern sensitive and confidential medical information.	specifying separate access to sensitive information for patients and parents, and embargoes on results delivered by providers.
The American College of Obstetricians and Gynecologists	2020	[88]	USA	comment	N/A	other stakeholders (HCPs)	pediatric obstetrics and gynecology	To provide recommendations for obstetrician–gynecologists and other HCPs who care for minors on confidential care for adolescents.	Authors made suggestions for protecting adolescent confidentiality, for example that there should be private conversation time between the HCP and the adolescent patient, and flagging portions of visit notes as "confidential".
Thompson et al.	2012	[47]	USA	quantitative	93	parents	outpatient (expectations)	To assess parents' current knowledge and opinions about technology facilitated HCP-adolescent communication.	The majority of parents disapproved of adolescents receiving private health information via secure web portals.
Thompson et al..	2016	[33]	USA	quantitative	adolescents, n=937 parents=963	adolescents (age 13-17.9) and parents	outpatient (experiences)	To explore how frequently adolescents will use a PAEHR that prioritizes adolescent confidentiality.	Adolescents: No gender differences in enrollment. Commonly used functions were test results, appointment review, and medications. Parents: High use. Commonly used functions were appointment review, messaging, and test results.
Tom et al.	2012	[49]	USA	quantitative	256	parents	outpatient (chronic disease) (experiences)	To explore how parents of children with chronic disease use PAEHRs and how users report experiences access to the health care teams.	Parents of children with chronic disease appear willing to use an integrated PHR to address health care needs for their child. Integrated PAEHR use was not associated with higher scores on CAHPS composite measures. Parents with public insurance were less likely to enroll.
Torrens et al.	2017	[39]	Australia	quantitative	N/A Not specified (2,074,800 registrations)	parents	non-clinical (population) (experiences)	To identify any subpopulations within the sex and age groups that were underrepresented in the registration pool.	Adolescents of both sexes were the least likely to register when compared with any other age group. In the age group 15-19, more females enrolled. Highest rates of activation among proxy users for age 0-4.
Weinstock	2018	[71]	USA	comment	N/A	other stakeholders (IT experts)	N/A Pediatric asthma (experiences)	To discuss an implemented PAEHR app and potential of PAEHRs.	It was stated that convenience and transparency of a PAEHR app was considered important by families.
Williams et al.	2016	[91]	USA	comment	N/A	other stakeholders (HCPs)	N/A adolescent	To uphold best ethical and legal practice on confidentiality especially in adolescent care.	Authors stated that education, advocacy, and research are required to ensure confidentiality of adolescent patients in their care. Also, more robust understanding of legal systems in which portal access is implemented will be required to uphold best practice in adolescent healthcare care.
Wong et al.	2017	[69]	New Zealand	comment	N/A	other stakeholders (HCPs)	N/A Non-clinical	To consider the legal and ethical framework to health information privacy and informed consent in	Authors proposed approaches to PAEHRs for young people. One proposal was that independent access for young people aged 16

								New Zealand, and propose an approach to implementing PAEHRs for young people.	years and over might be appropriate. An alternative proposal was shifting to independent access aged 12. Broader discussions about confidentiality, privacy, and parental involvement are recommended.
Wood et al.	2021	[34]	USA	quantitative	Not specified (merged sample of patient and proxy users, N=5862)	children/adolescents (age 12≤,)	pediatric inpatient, neonatal, intensive care (experiences)	To describe portal account activation among hospitalized children.	A total of 40.9% of patients had an activated account. Authors reported underuse of the patient portal in the inpatient pediatric population, especially for patients whose preferred language is not English, self-identify as multiracial and are publicly insured or uninsured.
Zellmer et al.	2021	[42]	USA	quantitative	parents = 25<br>HCPs = 25	parents and HCPs	pediatric inpatient care (experiences)	To solicit the views of parents and physicians on sharing notes with parents during hospitalization.	Parents all valued having access to physicians' notes during their child's hospital stay. HCPs saw benefits yet remained concerned about limiting documentation, increased workload, confusion for families, increased HCP stress, and reduced time at patient bedside.
